# Supplementary material for: The effect of non-linear signal in classification problems using gene expression
Source: PLoS Comput Biol. 2023 Mar 27;19(3):e1010984. doi: 10.1371/journal.pcbi.1010984 (PMC10079219; doi:10.1371/journal.pcbi.1010984)
Supplement: S1 Text — (DOCX) [file pcbi.1010984.s001.docx]

### S1 Text: Recount3 tissues used

The tissues used from Recount3 were blood, breast, stem cell, cervix, brain, kidney, umbilical cord, lung, epithelium, prostate, liver, heart, skin, colon, bone marrow, muscle, tonsil, blood vessel, spinal cord, testis, and placenta.
