# Supplementary figures and images for: The effect of non-linear signal in classification problems using gene expression
Source: PLoS Comput Biol. 2023 Mar 27;19(3):e1010984. doi: 10.1371/journal.pcbi.1010984 (PMC10079219; doi:10.1371/journal.pcbi.1010984)

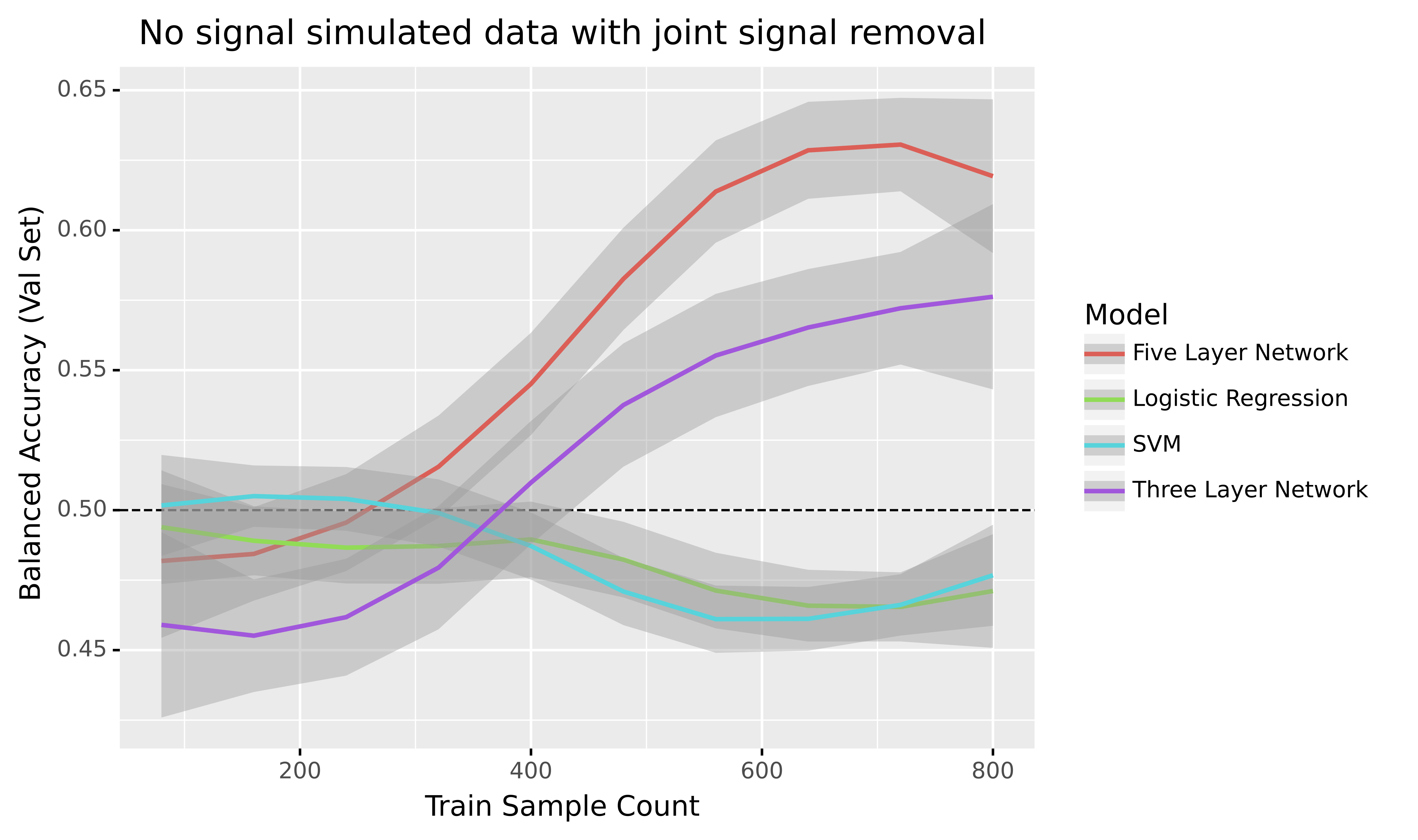

Supplement: S1 Fig — (TIFF) [file pcbi.1010984.s002.tiff]

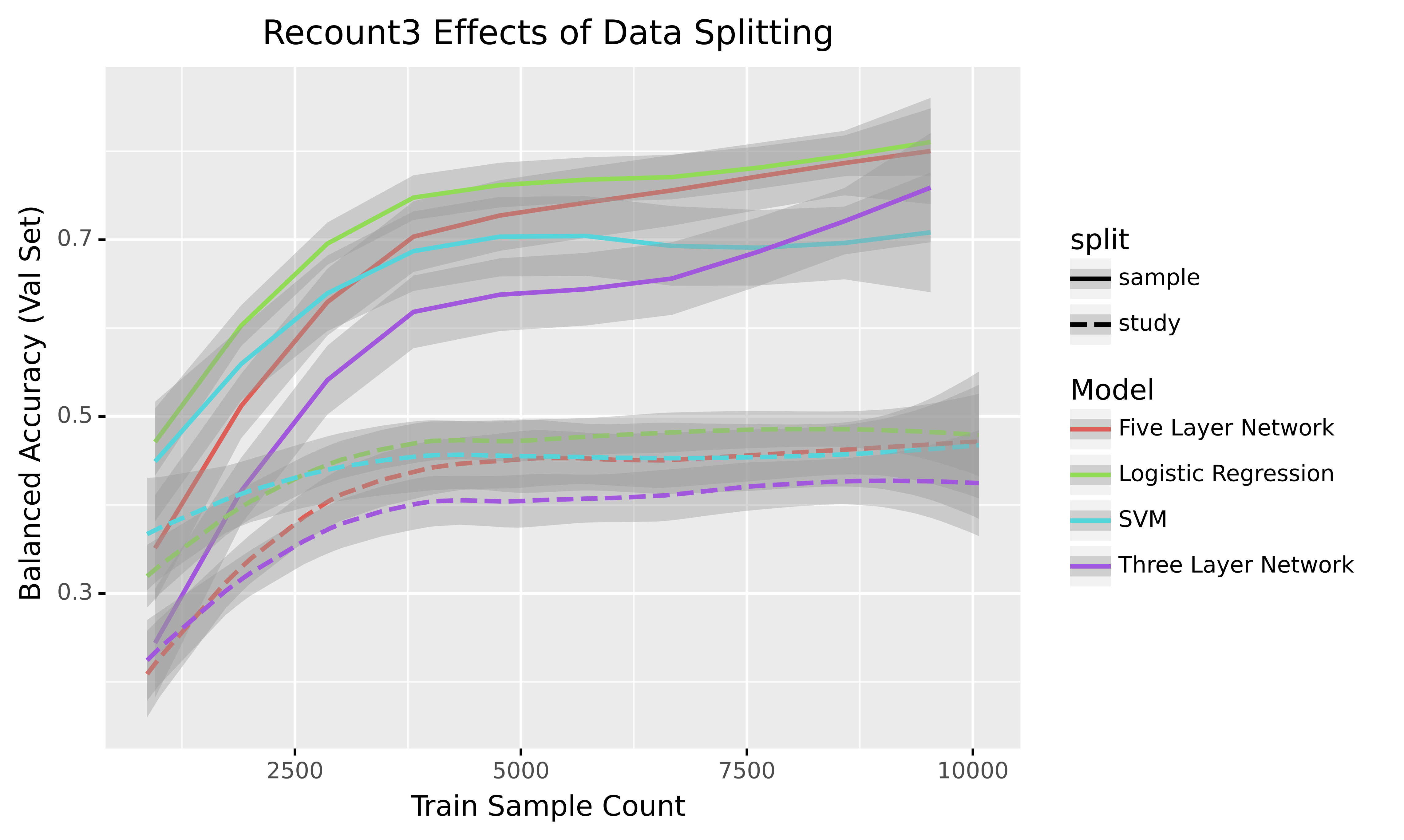

Supplement: S2 Fig — (TIFF) [file pcbi.1010984.s003.tiff]

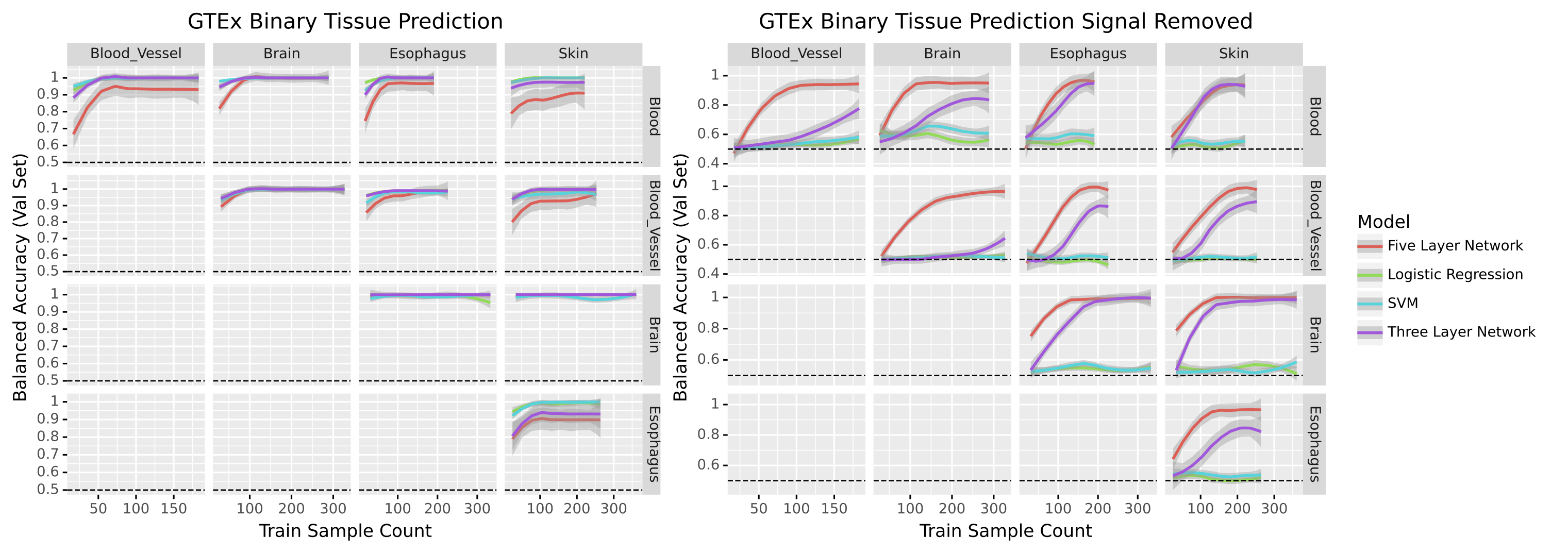

Supplement: S3 Fig — (TIFF) [file pcbi.1010984.s004.tiff]

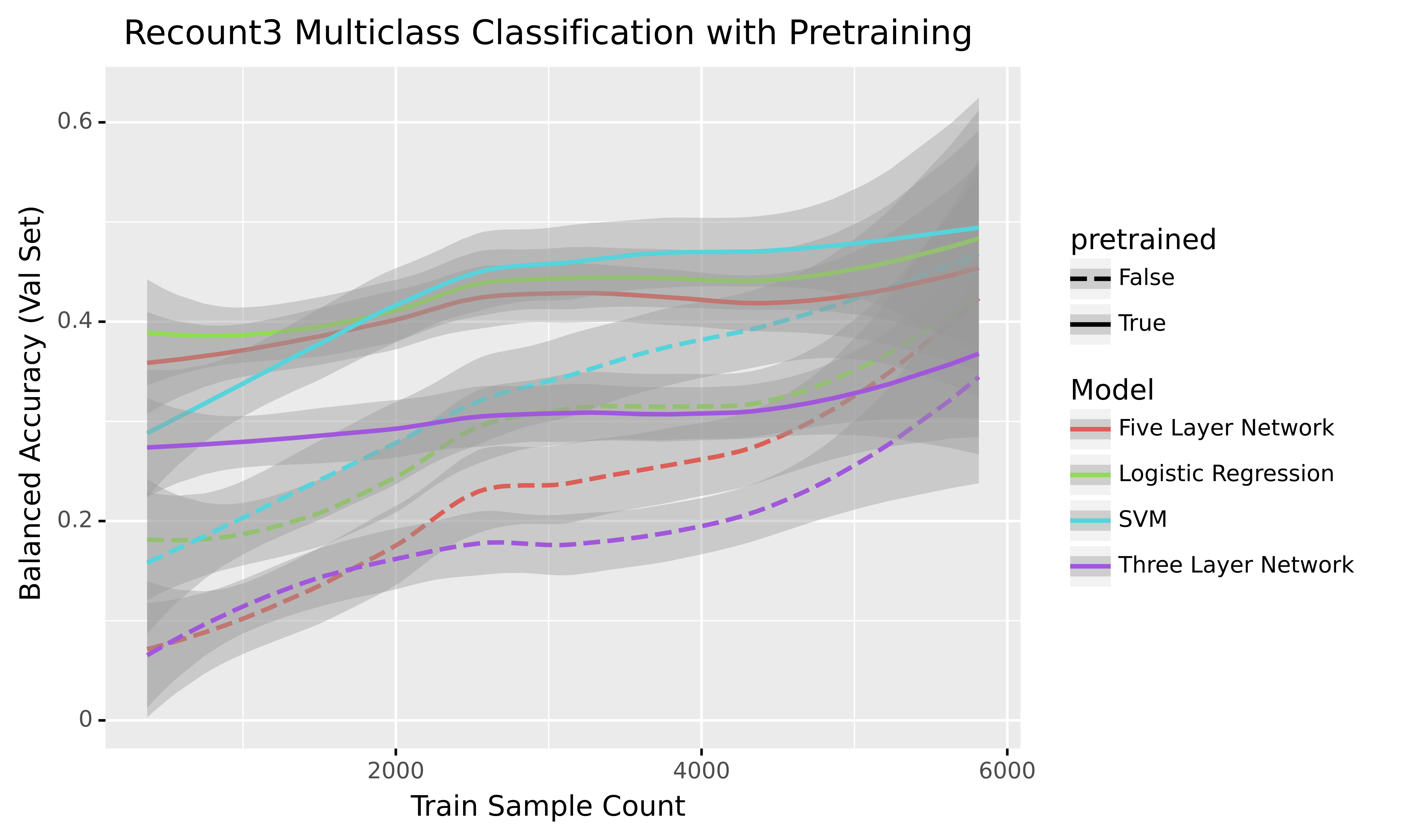

Supplement: S4 Fig — (TIFF) [file pcbi.1010984.s005.tiff]
