# Supplementary material for: The effect of non-linear signal in classification problems using gene expression
Source: PLoS Comput Biol. 2023 Mar 27;19(3):e1010984. doi: 10.1371/journal.pcbi.1010984 (PMC10079219; doi:10.1371/journal.pcbi.1010984)
Supplement: Attachment — Submitted filename: 128564_1_rebuttal_2477350_rj1f3y.pdf [file pcbi.1010984.s006.pdf]

Full Revision

Manuscript number: RC-2022-01574
Corresponding author(s): Casey, Greene

1. General Statements [optional]

We thank the reviewers for their thorough feedback. We have addressed all the points raised, revised the manuscript accordingly, and explained our changes below. To aid readability, the reviewers' comments have been converted to italics, and our responses have been bolded.

2. Point-by-point description of the revisions

Reviewer #1 (Evidence, reproducibility and clarity (Required)):

The authors systematically evaluate the performance of linear and non-linear ML methods for making predictions from gene expression data. The results are interesting and timely, and the experiments are well designed.

I have a few minor comments:
- It was hard for me to understand Figure 1B. I think a figure like this would be very helpful however. What do the numbers represent? If sample ID, then I am not sure why x-axis label is also "samples"
- For analysis of GTEx data, not sure what "studywise splitting" would mean, since the GTEx dataset is one study? Do you leave out the same individuals from all tissues for evaluation?

We thank the reviewer for their input on these two points. To make Figure 1B clearer and to elaborate on our stratified splitting methods, we have amended its description to "We stratify the samples into cross-validation folds based on their study (in Recount3) or donor (in GTEx). We also evaluate the effects of sample-wise splitting and pretraining (B)."

- I found the sample size on x-axis of Fig 2a confusing. If I understand correctly, GTEx has a total of ~1000 subjects. So in some sense, effective sample size can not be bigger than 1000. If you are counting subjects x tissue as sample, then it can be misleading in terms of the effective sample size.

We thank the reviewer for this point. To incorporate it into the manuscript, we've added the following text to the description of Fig. 2: "It is worth noting that "Sample Count" in these figures refers to the total number of RNA-seq samples, some of which share donors. As a result, the effective sample size may be lower than the sample count. "

Review COMMONS

- Would be interesting to assess out-of-sample generalizability of linear and non-linear models. Have you tried training on GTEx and predicting on Recount3 or vice versa?

This question intrigued us. We reran the tissue prediction experiments from the manuscript on a subset of the GTEx and Recount3 datasets in which we performed an intersection over tissues and genes. We found that in the out-of-sample domain the logistic regression model and the three layer neural network performed similarly, while the five layer net generally had a lower accuracy despite having similar accuracy in the training domain. We also found (consistent with our results in the paper) that GTEx predictions are an easier task than their Recount counterparts. Below are plots demonstrating these findings:

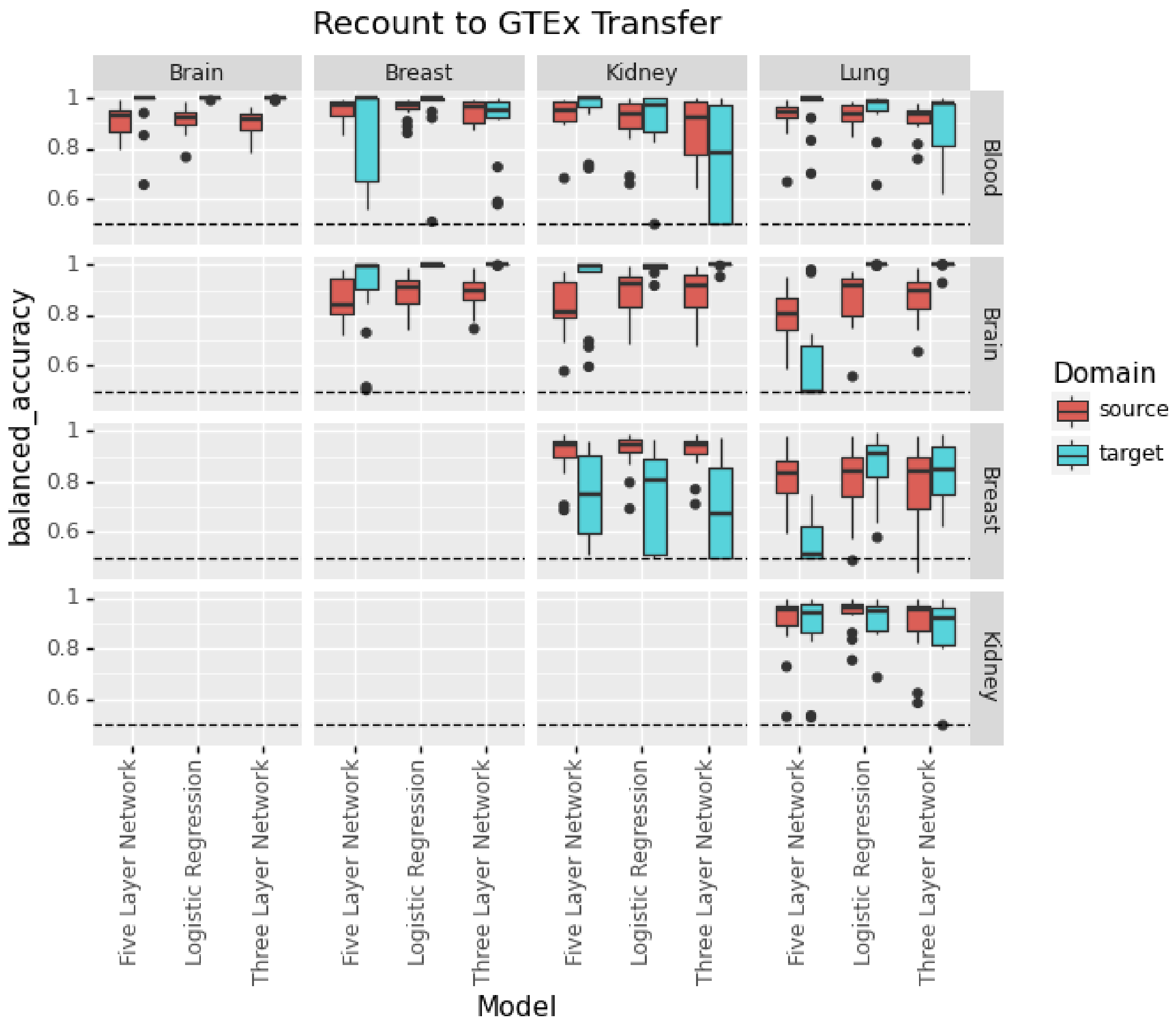

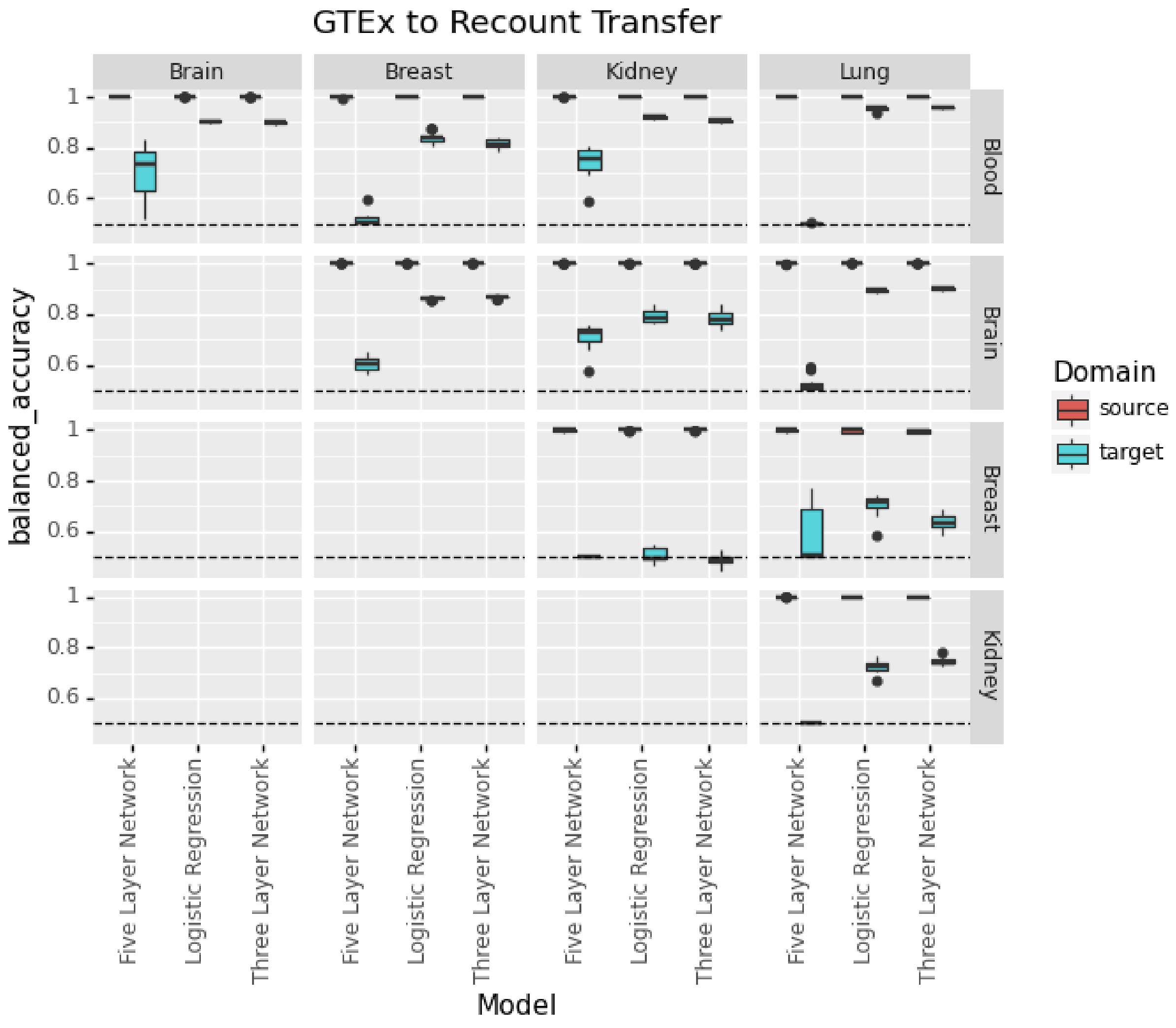
GTEx to Recount Transfer
Brain
Breast
Kidney
Lung
Blood
Brain
Breast
Kidney
1
0.8
0.6
balanced_accuracy
Domain
source
target
Five Layer Network
Logistic Regression
Three Layer Network
Model

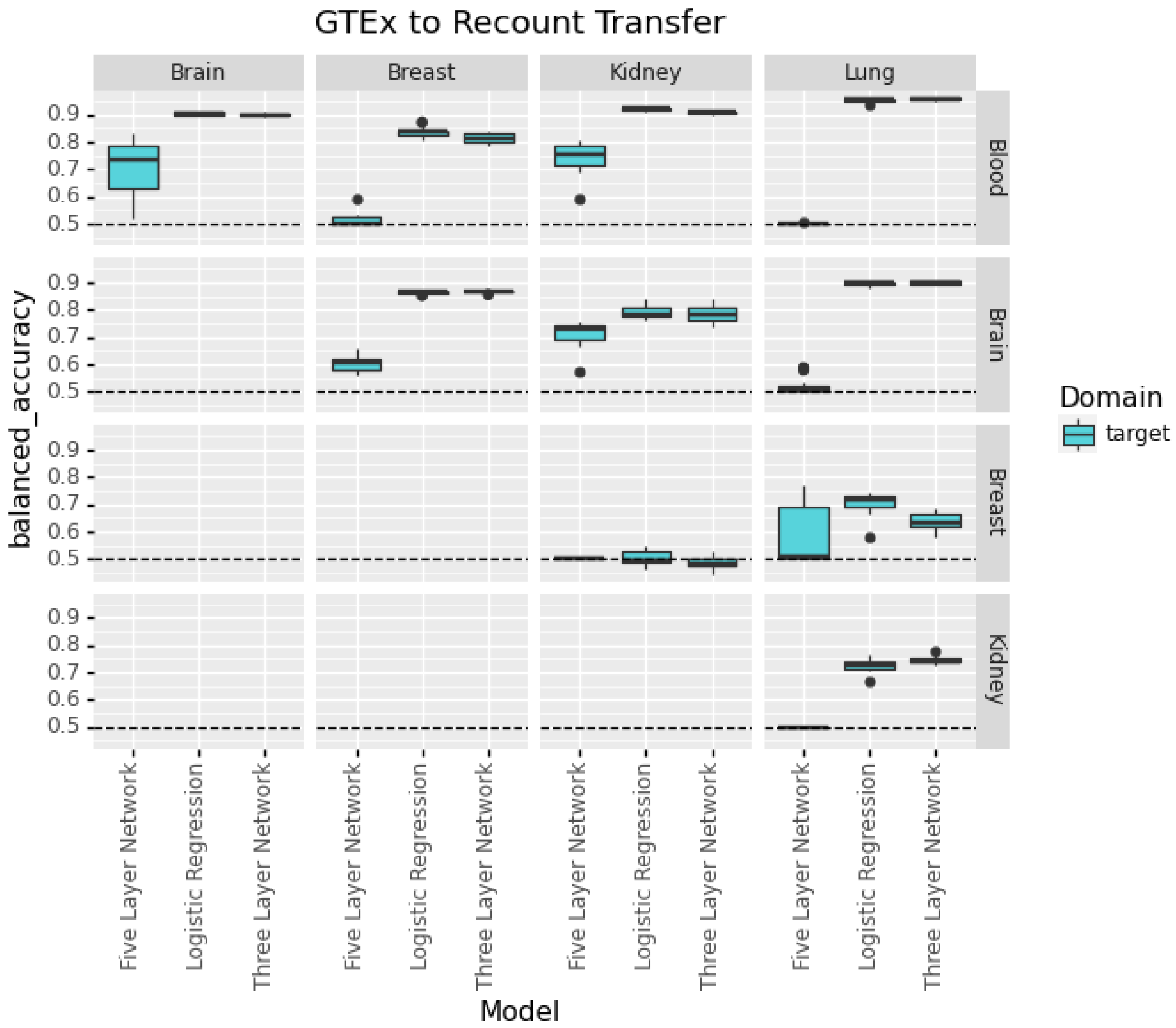
GTEx to Recount Transfer
Brain
Breast
Kidney
Lung
Blood
Brain
Breast
Kidney
balanced_accuracy
0.9
0.8
0.7
0.6
0.5
Domain
target
Five Layer Network
Logistic Regression
Three Layer Network
Model

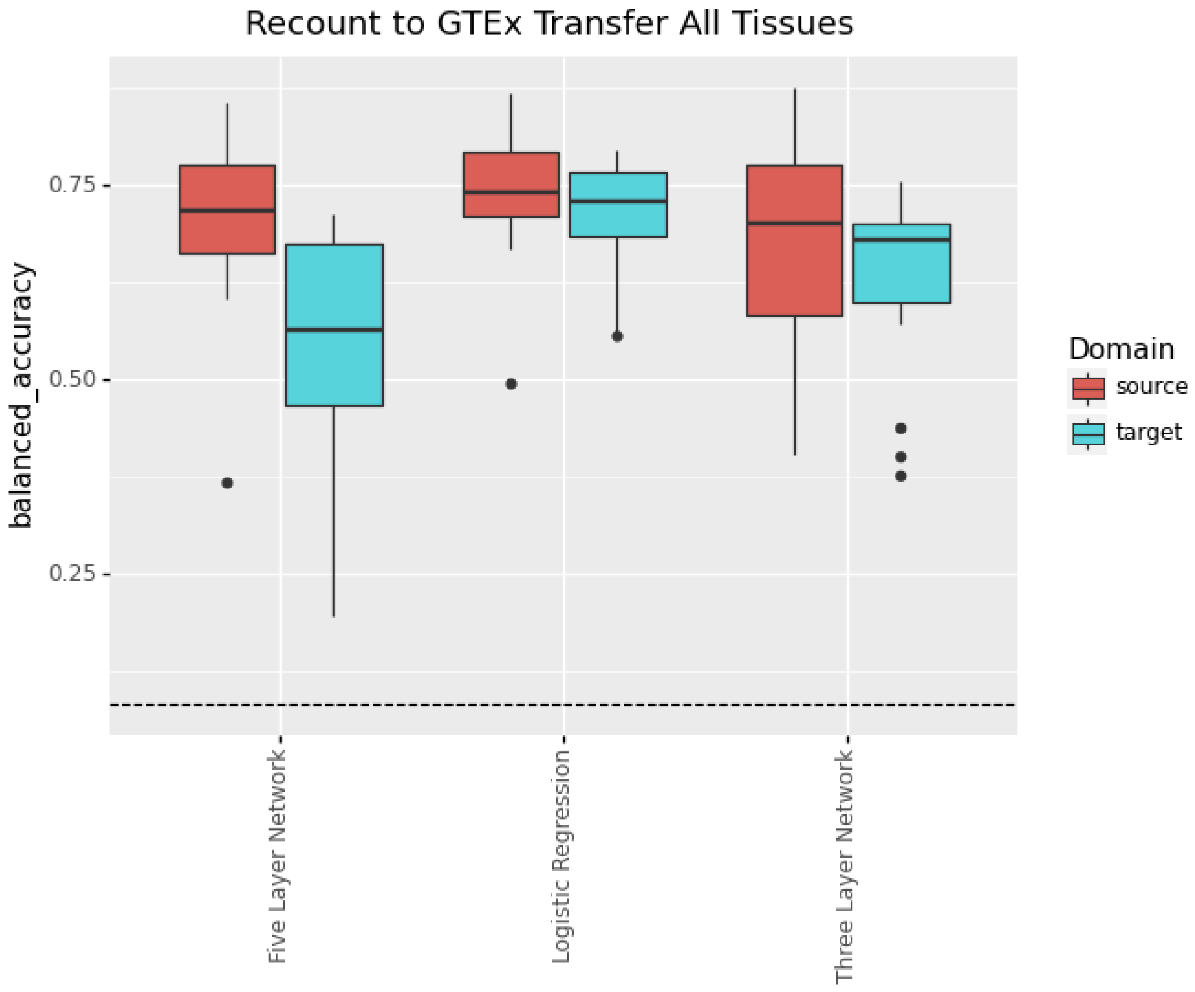
Recount to GTEx Transfer All Tissues
balanced_accuracy
0.75
0.50
0.25
Five Layer Network
Logistic Regression
Three Layer Network
Model
Domain
source
target

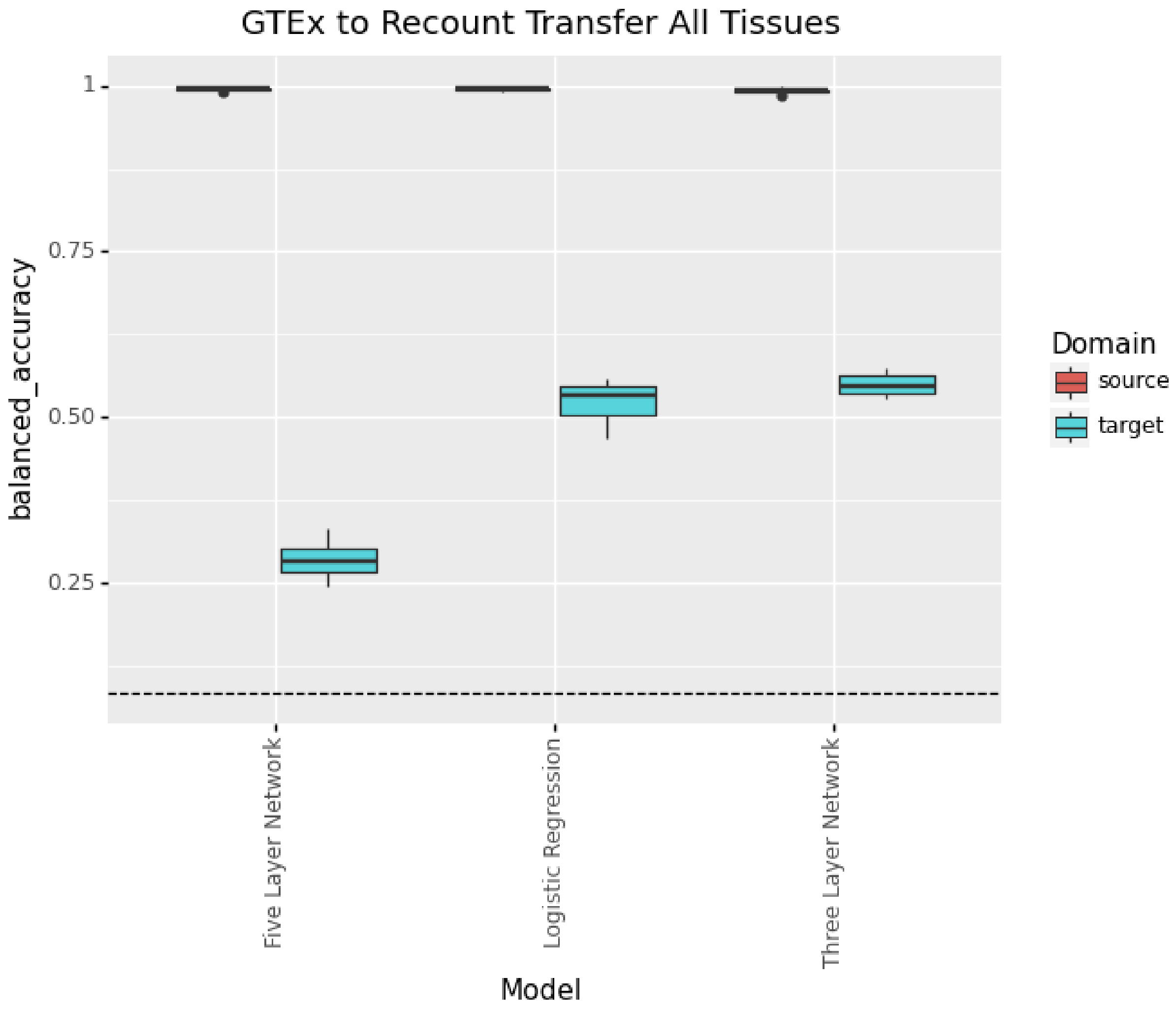

Reviewer #1 (Significance (Required)):

Important and timely study, evaluating linear vs non-linear methods for predicting phenotype from gene expression datasets.

We appreciate the reviewer's positive comments on the timeliness of our manuscript.

Reviewer #2 (Evidence, reproducibility and clarity (Required)):

Summary

The authors want to assess the presence of non-linear signal in gene expression values in the task of tissue and sex classification. They use logisitic regression classifiers and two types of

Review COMMONS

neural networks, with 3 and 5 layers, and assess classification performance on two large expression datasets from Recount3 and GTEX and three simulated datasets.
The authors carefully construct their learning setup in such a way that one can reason about the removal of linear signal from the expression features. The interesting conclusion is, that although the linear approach works well on both datasets, and sometimes even better than the more complex models. The authors convingly show, that there is a significant non-linearity in the gene expression data. However, just because it is "there" does not imply that any non-linear methods performs better.

Major comments:
- Are the key conclusions convincing?
The authors did a good job in showing, that there is non-linear signal in gene expression features for the classification problems studied.

We thank the reviwer for their positive feedback.

- Should the authors qualify some of their claims as preliminary or speculative, or remove them altogether?

The overall claims of the authors are justified, the discussion may be improved.

We appreciate the reviewer's support for our overall claims and we have adjusted the manuscript as noted point by point below.

- Would additional experiments be essential to support the claims of the paper?

No, additional experiments are not essential. But the authors did not compare to other non-linear methods such as SVM or knn-classifiers in the resulst or conclusion section. It is unlikely that the main conclusion would change if those methods were tried. But it is possible that other "simpler" non-linear methods, such as knn for example, are able to outperform the logistic regression classifier on the GTEX and Recount3 data set. Thus, the authors should at least mention this as part of the conclusion and could extend their discussion on the implications of their study concerning other tasks or models.

We agree that there should be more discussion of other models in the conclusion section. We have updated the fifth paragraph of the conclusion accordingly:
"We are also unable to make claims about all problem domains or model classes. There are many potential transcriptomic prediction tasks and many datasets to perform them on. While we show that non-linear signal is not always helpful in tissue or sex prediction, and others have shown the same for various disease prediction tasks, there may be problems where non-linear signal is more important. It is also possible that other classes

Review COMMONS

of models, be they simpler nonlinear models or different neural network topologies are more capable of taking advantage of the nonlinear signal present in the data."

- Are the suggested experiments realistic in terms of time and resources?
Not applicable.

- Are the data and the methods presented in such a way that they can be reproduced?

There is a separate github repo which has the code to reproduce the analyses. This is good. However, would be nice to explain in more detail in the manuscript how the limma function was used for removing the linear signal, as they mention the "removeBatchEffect" function was used, but it would be good to tell the reader how that works, as this is their way for assessing the effect of linear-signal removal. Are there any limitations for the assessment of signal removal in this way?

We thank the reviewer for their input, and have updated the model training section on signal removal to read: "We also used Limma[24] to remove linear signal associated with tissues in the data. We ran the 'removeBatchEffect' function on the training and validation sets separately, using the tissue labels as batch labels. This function fits a linear model that learns to predict the training data from the batch labels, and uses that model to regress out the linear signal within the training data that is predictive of the batch labels."

We have also elaborated on the limitations of signal removal by updating the sentence "This experiment supported our decision to perform signal removal on the training and validation sets separately, as removing the linear signal in the full dataset induced predictive signal (supp. fig. 6)" to read "This experiment supported our decision to perform signal removal on the training and validation sets separately. One potential failure state when using the signal removal method would be if it induced new signal as it removed the old. This state can be seen when removing the linear signal in the full dataset(supp. fig. 6)."

- Are the experiments adequately replicated and statistical analysis adequate?

Yes

Minor comments:
- Specific experimental issues that are easily addressable.
no

- Are prior studies referenced appropriately?
Yes
- Are the text and figures clear and accurate?

Also, they conducted 3 different experiments in Figure 3. It would be useful to separate the figure into 3) A, 3) B, and 3) C and link that specifically in the text. Figure 4 is an extended version of Figure 2, just with the additional results of the signal removed performances.

We appreciate the feedback. To make the figure and the text more clear, we have added A, B, and C subheadings to figure 3, and updated the subfigure's references within the text accordingly.

First, the pairwise results in 4B are hard to read as the differences in colors and line type are difficult to see as some lines are short. Second, we did not find it helpful to reproduce the full signal approach in Figure 4. We would suggest to make Figure 4 as Figure 2, and simply only talk about the Full signal mode in the beginning, how it is in the text.

We agree. We have made Figure 4 our new Figure 2 and updated the references in the text.

Further, it would be nice to give better names in the legends of these plots. Pytorch_lr is not a nice name.

We thank the reviewer for pointing this out. We have updated the names in the legends to be "Five Layer Network", "Three Layer Network", and "Logistic Regression"

- Do you have suggestions that would help the authors improve the presentation of their data and conclusions?

As the Recount3 dataset is different in quality and complexity it would be reasonable to show the results of the binary classifcation also in the main paper. In particular, as this behaves different to the GTEX binary classification.

We have now moved the Recount binary classification figure from the supplement to join the GTEx binary classification data as the new figure 4.

-The title is somewhat unprecise. It may induce the impression that the paper is about expression-prediction, although that is not the case. Further, in the abstract they don't mention what prediction problem they solve and that these are classification problems. After reading the paper it is clear why the authors choose that, but we are suggesting an alternative title that the authors may consider:

The effect of nonlinear signal in classification problems using gene expression values

We agree with the reviewer's comment and have updated our title to "The effect of non-linear signal in classification problems using gene expression"

Review COMMONS

Further, they should give more details on the problem learned in the abstract.

We thank the reviewer for their feedback, and have added details to the abstract about the problem domains. The relevant sentence now reads “We verified the presence of non-linear signal when predicting tissue and metadata sex labels from expression data by removing the predictive linear signal with Limma, and showed the removal ablated the performance of linear methods but not non-linear ones.”

-In addition, the conclusion section, which may be title as Disucssion and Conclusion, could contain additional points concerning the topology and training of the neural networks.

We have updated the heading of the final section to Discussion and Conclusion. To expand on the potential drawbacks of our neural network topologies, we have also updated the limitation portion of Discussion and Conclusion to read “We are also unable to make claims about all problem domains or model classes. There are many potential transcriptomic prediction tasks and many datasets to perform them on. While we show that non-linear signal is not always helpful in tissue or sex prediction, and others have shown the same for various disease prediction tasks, there may be problems where non-linear signal is more important. It is also possible that other classes of models, be they simpler nonlinear models or different neural network topologies are more capable of taking advantage of the nonlinear signal present in the data.”

Obviously, it is possible that other simpler or more complex neural networks have a better performance on the GTEX and Recount3 data sets compared to logistic regression. In fact, the results from Figure4 suggest that, as there is clearly useful non-linear signal in those datasets for the classification problems studied. However, optimizing a non-linear model is inherently more complex and time-consuming, and thus may not be done thoroughly in previously published papers. Compared to a linear model that is easier and faster to optimize, this may be one reason why studies find that, despite non-linear signal, the linear model performs better. Other factors such as the samples size, which the authors already mention, of course also plays a big role, and if hundreds of thousands of datasets would be there , e.g. from single cell measurements, non-linear methods may have a better chance of outcompeting linear models.

We agree, which is why we consider the signal removal experiment to be so important. By demonstrating that the non-linear methods we used were in fact learning non-linear signal we were able to show that there was something that non-linear models were able to learn that logistic regression was unable to. That is to say that while the presence of non-linearity in the decision boundary is necessary for non-linear models to outperform linear ones, it is not by itself sufficient. Perhaps with more data or a different model non-linear methods would perform better, but there is certainly a class of models and problems where logistic regression is preferable.

Reviewer #2 (Significance (Required)):

The submitted manuscript adds to the discussion of the necessity of non-linear models when solving classification problems using gene expression data. The significance is mostly technically, as a comparison of logistic regression and two neural network topologies that are being compared on two large expression datasets. However, there is also a conceptual part of the contribution, which is with regards to the implications of their experiments.
Interested audience would be computer scientists and bioinformaticians or others, that are involved in creating or interpreting these or similar prediction models.

Our field of expertise is in the creation of machine learning models using different types of OMICs data. All aspects of the work could be assessed.

Reviewer #3 (Evidence, reproducibility and clarity (Required)):

In this manuscript, the authors discuss an interesting problem regarding the comparative performance of linear and non-linear machine learning models. The main conclusion is that logistic regression (linear model) and neural networks (non-linear model) have comparable performance if the data contain both linear and non-linear relations between the features (X) and the prediction target (Y), however, if the linear component in the X-Y relation is removed (e.g. regressed out) the neural networks will outperform logistic regression. This conclusion implies that linear models such as logistic regression mainly relies on the linearity in the X-Y relation.
However, whether X-Y relation has a linear component and whether the data (e.g. for different Y classes) are linearly separable are two different questions. For example, consider a data generating mechanism, y=x^2+x and label the data points using two classes (y<=1 and y>1). Clearly, the data is linearly separable, and any machine learning algorithm should perform very well on this problem. Now remove the linear component form the X-Y relation and use y=x^2 to generate the data. The data is still linearly separable, and the performance of logistic regression should not be affected.

We agree that there is a difference between optimal linear decision boundaries and linear relationships between elements in the training data. Our use of the term "relationship" in place of "decision boundary" was imprecise. To make this more clear, we have made the following changes:

Introduction:
"Unlike purely linear models such as logistic regression, non-linear models should learn more sophisticated representations of the relationships between expression and

phenotype." -> "Unlike purely linear models such as logistic regression, non-linear models can learn non-linear decision boundaries to differentiate phenotypes."

"However, upon removing the linear signals relating the phenotype to gene expression we find non-linear signal in the data even when the linear models outperform the non-linear ones." -> "However, when we remove any linear separability from the data, we find non-linear models are still able to make useful predictions even when the linear models previously outperformed the nonlinear ones."

Discussion and conclusion:
We removed the following paragraph: "Given that non-linear signal is present in our problem domains, why doesn't that signal allow non-linear models to make better predictions? Perhaps the signal is simply drowned out. Recent work has shown that only a fraction of a percent of gene-gene relationships have strong non-linear correlation despite a weak linear one [23]."

The point is that the performance of linear models is mainly dependent on whether the data are linearly separable instead of the linearity in X-Y relation as the manuscript suggests.

We agree that this is the key point and appreciate the reviewer for helping us to more carefully hone the language to convey this point.

Reviewer #3 (Significance (Required)):

The performance comparison between linear and non-linear machine learning models is important.

We appreciate the reviewer's recognition of the significance of the work.
