# Supplementary material for: The effect of non-linear signal in classification problems using gene expression
Source: PLoS Comput Biol. 2023 Mar 27;19(3):e1010984. doi: 10.1371/journal.pcbi.1010984 (PMC10079219; doi:10.1371/journal.pcbi.1010984)
Supplement: Attachment — Submitted filename: linear_models_response_to_reviewers_2.pdf [file pcbi.1010984.s007.pdf]

We thank the reviewers for their thorough feedback. We have addressed all the points raised, revised the manuscript accordingly, and explained our changes below. To aid readability, the reviewers' comments have been converted to italics, and our responses have been bolded.

Reviewer #1: *In this resubmitted manuscript, the authors systematically compare multi-layer neural networks and logistic regression across multiple prediction tasks on GTEx and Recount3 datasets. The authors verified the presence of non-linear signal when predicting tissue and metadata sex labels from expression data by removing the predictive linear signals. The authors also showed that the presence of non-linear signal was not necessarily sufficient for neural networks to outperform logistic regression. Finally, the authors argued that while multi-layer neural networks might be useful for making predictions from gene expression data, including a linear baseline model is critical.*

The paper is very well-written and easy to follow. The experiments are well designed, and the different stratification strategy of the data splitting is thoughtful. I don't have any comments because they have been already well addressed in the response!

We thank the reviewer for their kind words and their appreciation of our stratification strategy!

Reviewer #2: *The authors try to evaluate the performance of linear and non-linear models in the presence and absence of linear signals. Logistic Regression was taken as a representative of linear models whereas a three-layered and a five-layered neural network were considered as non-linear models. Based on the experimental results in binary and multi-class classification tasks on the GTEx, Recount3, and three simulated datasets, the authors claim that the presence of task-related non-linear signals in the data does not necessarily assist the non-linear models to outperform the linear ones. Although the authors focus on an interesting topic and perform several experiments, still a lot more experiments with varying factors needs to be done before reaching any reasonable conclusion.*

Strengths:

• Through experimentation by removing the linear signals, the authors prove that the similarity in the performance of the different models was not due to the sole presence of linear signals.

We thank the reviewer for pointing out this strength. The presence of nonlinear signal despite similar performance across models is not something we had seen before either in gene expression studies or in the broader context of machine learning.

• Assessment of the models' performance was also done with pretraining where the balanced accuracy was similar across the model.

We thank the reviewer for their positive feedback!

• Experiments with sample-wise splitting and study-wise splitting better justify the results and uphold the claim of the authors.

We appreciate the reviewer noticing this point. We feel that sample splitting could often be improved in applications of machine learning to gene expression data.

Drawbacks:

• It would be interesting to assess the models' performance in the case of small imbalanced datasets with high dimensionality which is often the case in biological data.

We thank the reviewer for their comment and agree that class imbalance is very prevalent in biological data. In fact, our multiclass setting included tissues with as many as 5644 samples (blood), and as few as 48 samples (testis) (a 100:1 imbalance), while the binary tasks had a 10:1 imbalance in e.g. blood vs stem cells. Our datasets were also high-dimensional, with $p > n$ in many cases, such as the binary tasks where there were 5000 genes used to train models on only hundreds of samples.

The reviewer raises a good point that the class-imbalance and dimensionality aspects of our analyses are not as well-discussed as would be ideal. To address this shortcoming, we've added a paragraph to the results section that reads "It is worth noting that the relative performance of the models remain the same despite class imbalances and different ratios of genes to samples. For example, the ratio of genes to samples goes all the way from roughly 10:1 in the heavily subsetted binary classification regime to 1:4 in the full sex label prediction dataset."

We've also added a supplementary figure (supp. fig. 9), and the following text to the results section "We also ensured that these results were also applicable at small sample sizes by running the GTEx binary prediction tasks with the training dataset reduced to 1-10% of its original size. We continued to see similar performance across model classes, though the accuracies were lower across the board due to the decreased training data"

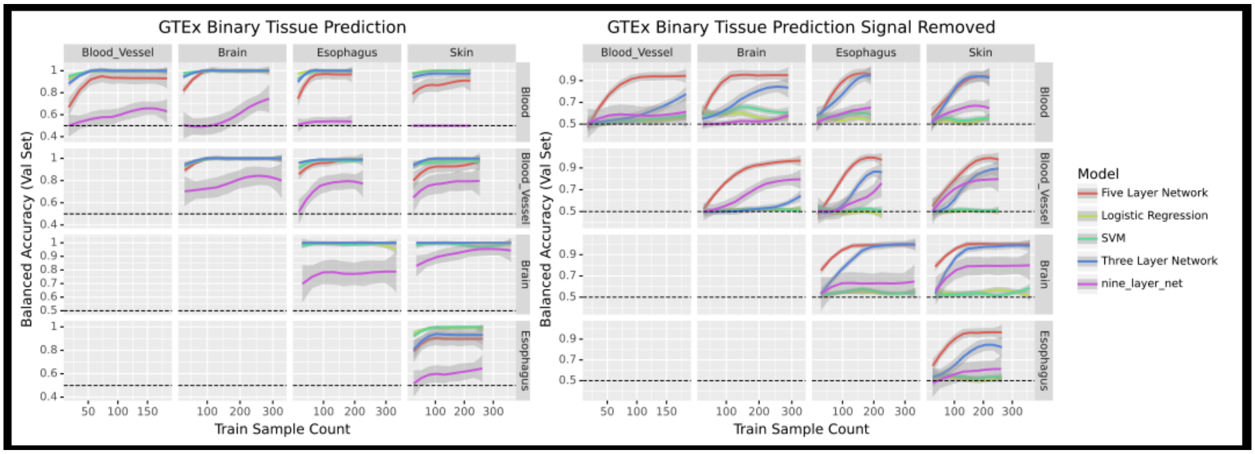

• *More sophisticated linear models like- SVM could be used for comparison. Similarly, deeper models with hundred layers would be better representative of non-linear models.*

We agree that adding models to our analysis would increase its representativity. To that end, we implemented a linear SVM in Pytorch and found that it performs similarly to logistic regression in most cases. We then updated the model comparison figures and added descriptions of the model's performance to the relevant sections of the results.

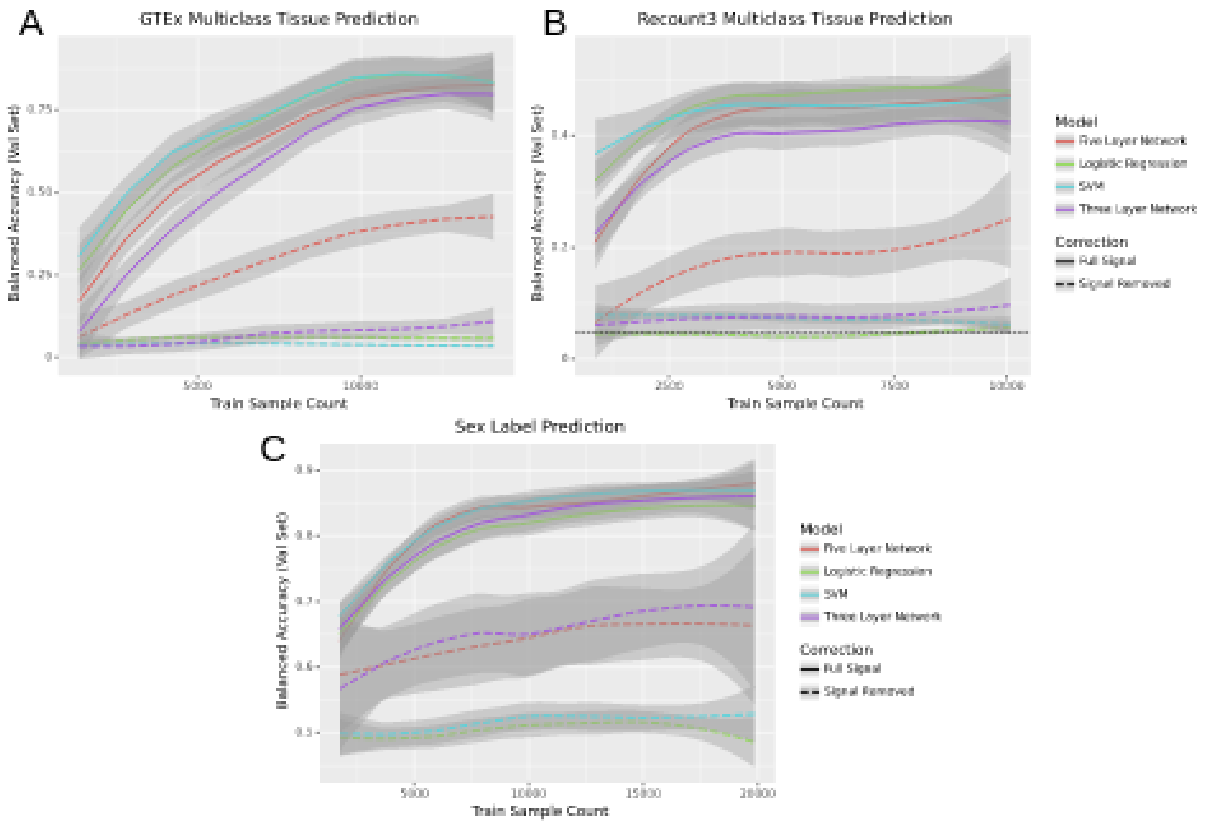

While deep learning is popular, deeper models are more challenging to train both technically and in terms of the amount of computation required. We began the training of nine layer networks for the tasks in the paper, but found that they consistently underperformed the other models (as seen below). As the wall-time required to run all the nine-layer tasks on our HPC and the local hardware available to us would be longer than the two months allotted for revisions, we elected not to include the nine layer network in the manuscript.

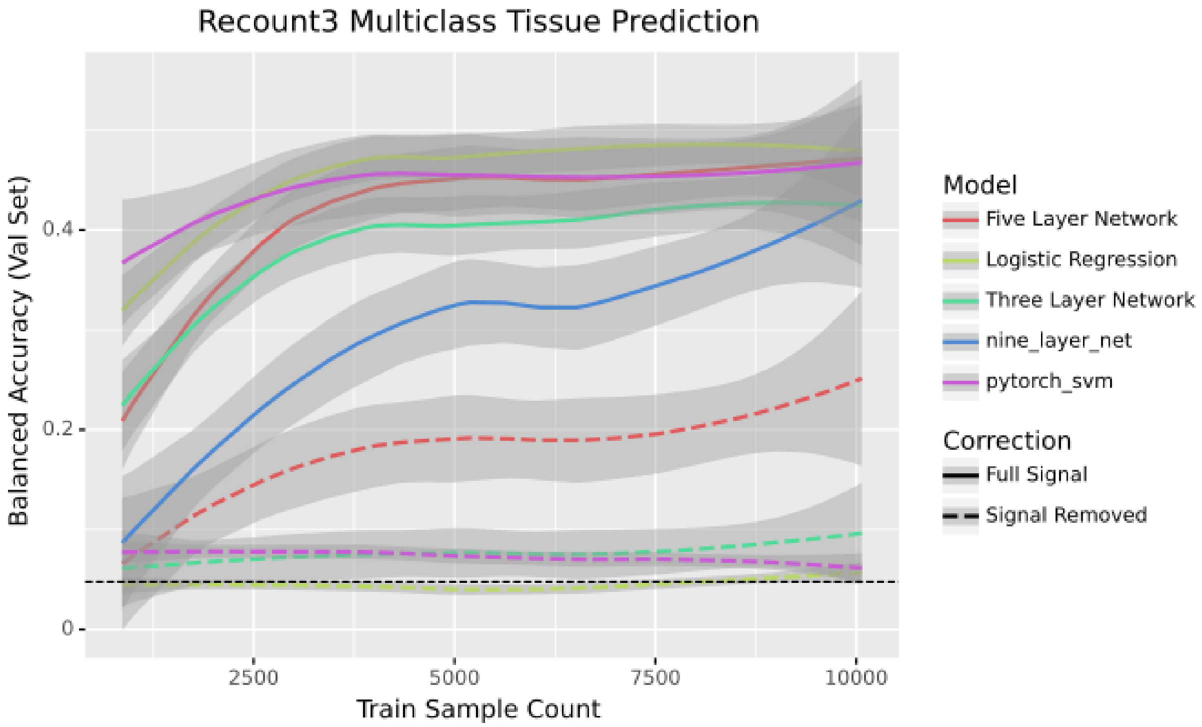

• *Experiments by varying the number of genes for the specific tasks would be helpful in the evaluation of the authors' claim.*

We appreciate the reviewer's suggestion. To evaluate the effect the number of genes had on our models we generated simulated data with varying numbers of genes. Upon doing so we found that the number of genes did not have a consistent effect on balanced accuracy across models. Because we were unable to determine what if any effect genes had, we elected not to include the results in the manuscript. Examples of what we saw are below:

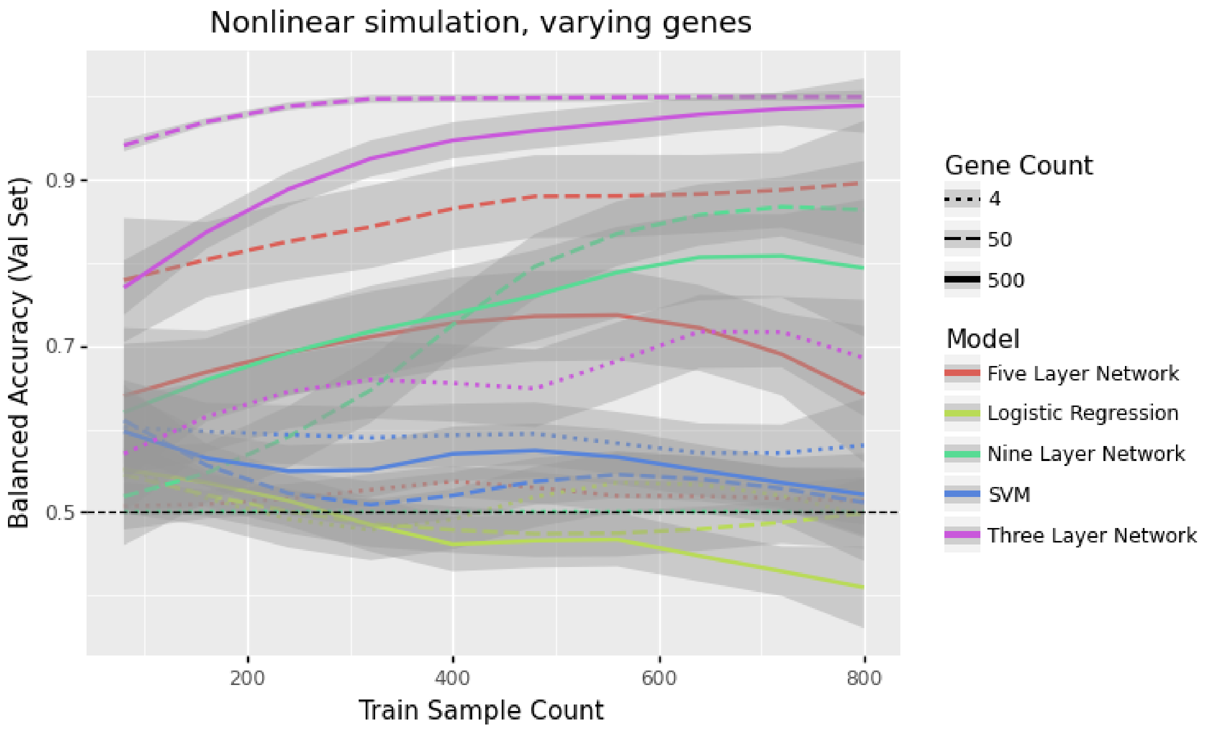

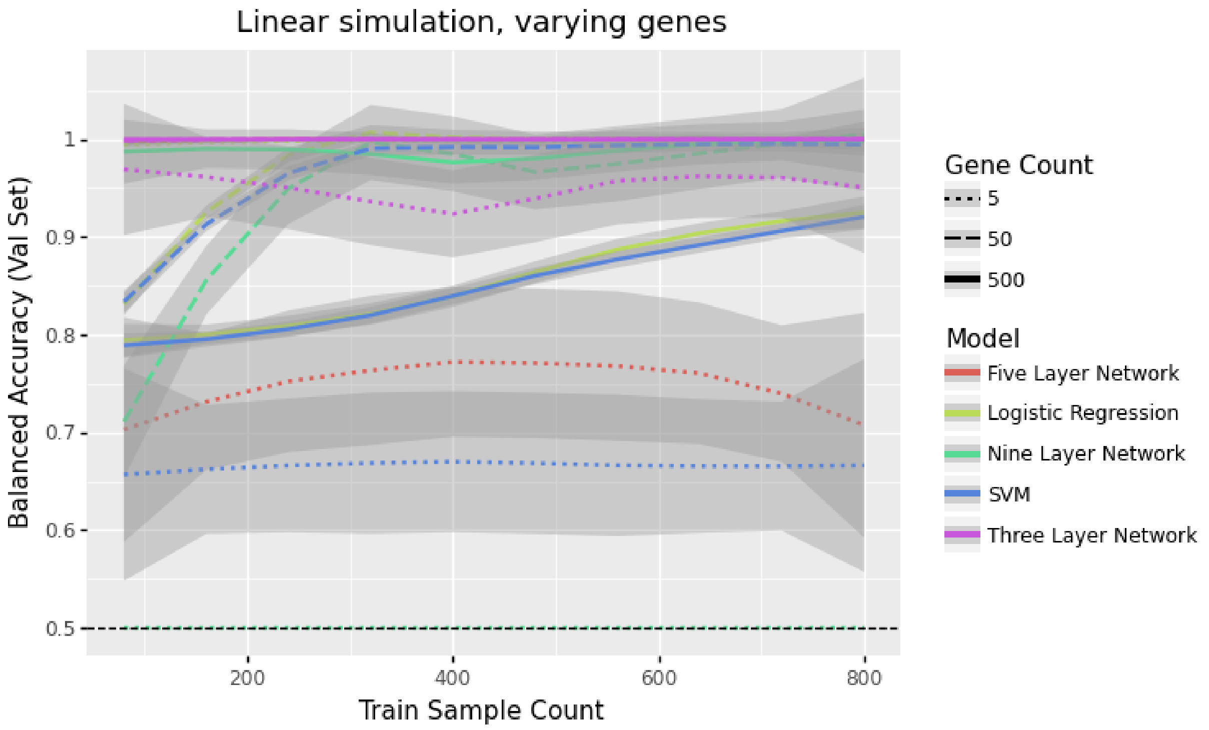

Reviewer #3: Some suggestions and questions:

1. Although the reviewers in the previous round pointed it out, I am still confused by the number in Fig 1B.

We appreciate the reviewer's feedback! As the figure was leading to confusion, we have updated the description, adding the sentence "Each circle in the figure represents a sample, which is numbered according to its study."

2. Figure numbers are not cited in the order. For example, Fig4A appeared before Fig2 and 3.

In the revised manuscript we have rearranged the figures so that their corresponding numbers are now referenced in order.

3. Above Figure 2, there are three words "TODO update description." What are they?

We thank the reviewer for pointing out this artifact. It was a holdover from a previous version of the manuscript that we have now removed.

4. There is no supp. fig.4B, but it was cited above the Figure 2.

We appreciate the reviewer for pointing this out; the reference was supposed to be to fig. 4B, not supplementary figure 4B. We have fixed this in the revised manuscript.

5. Why does the logistic regression perform much worse than the other two non-linear models under the linear simulation scenario (Figure 3A)? Also, the logistic regression model performs similarly for the linear and non-linear simulation (Figures 3A and B). Is it a little bit counterintuitive?

We believe that the decrease in performance is caused by the high degree of redundancy of the features in the simulated data. While the neural networks use a dropout regularization step that allows them to avoid focusing on individual features to too great a degree the linear models do not use dropout.

For the second part, we realized that our terminology was unclear. What we termed "nonlinear" would be more accurately called "linear + nonlinear signal" because we are adding nonlinear features to data that still contains the linear ones. Because the linear models should be unable to learn meaningful correlations from the nonlinear features in the nonlinear simulation, we expect them to instead learn from the linear features in the nonlinear simulation data (the nonlinear simulation data contains 2500 features with linear signal as well as the 2500 features with nonlinear signal). Because the linear features are generated in the same way in both the linear and nonlinear datasets, the models' ability to fit the datasets is very similar. We apologize for the confusing nomenclature in the original submission.

6. In the last sentence before the Methods section: "However, the non-linear models did not have a greater performance gain from pretraining than logistic regression, and the balanced accuracy was similar across models." If it is referring to supp.fig.8, I did see a big improvement for the non-linear model, and balanced accuracy definitely varies across models. I am curious how did the authors get this conclusion?

We thank the reviewer for pointing out a lack of clarity in our writing. Our intent was to demonstrate that while pretraining can be helpful, it's not *more* helpful for nonlinear models. Supp. fig. 8 demonstrates this by showing similar gains in accuracy between the linear and nonlinear models.

To alleviate the confusion, we've edited the sentence to read, "The pretrained models showed a gain in performance over the non-pretrained models even when trained with small amounts of data from the training set. However, the non-linear models did not have a greater increase in performance from pretraining than logistic regression, and the balanced accuracy was similar across pretrained models."
